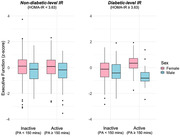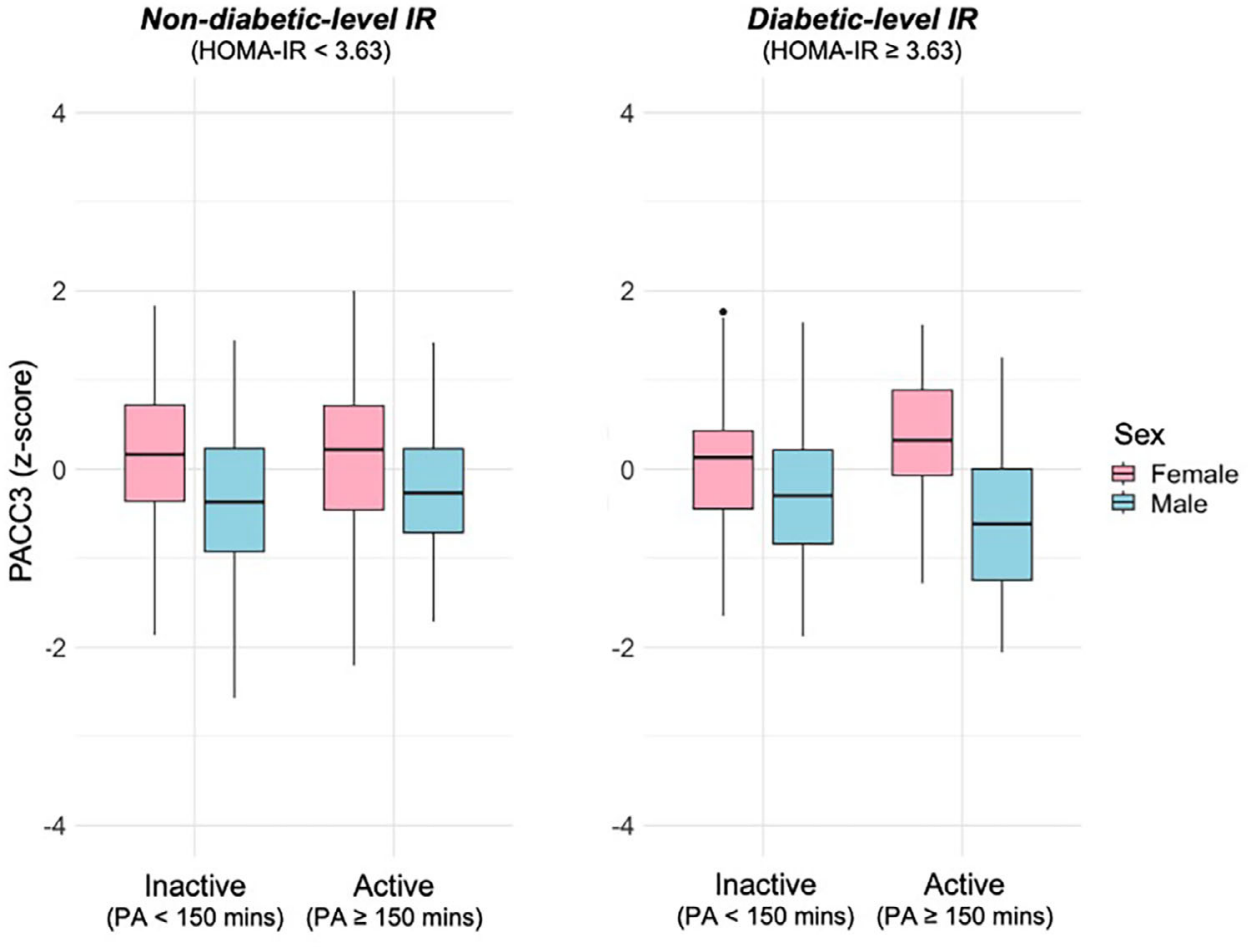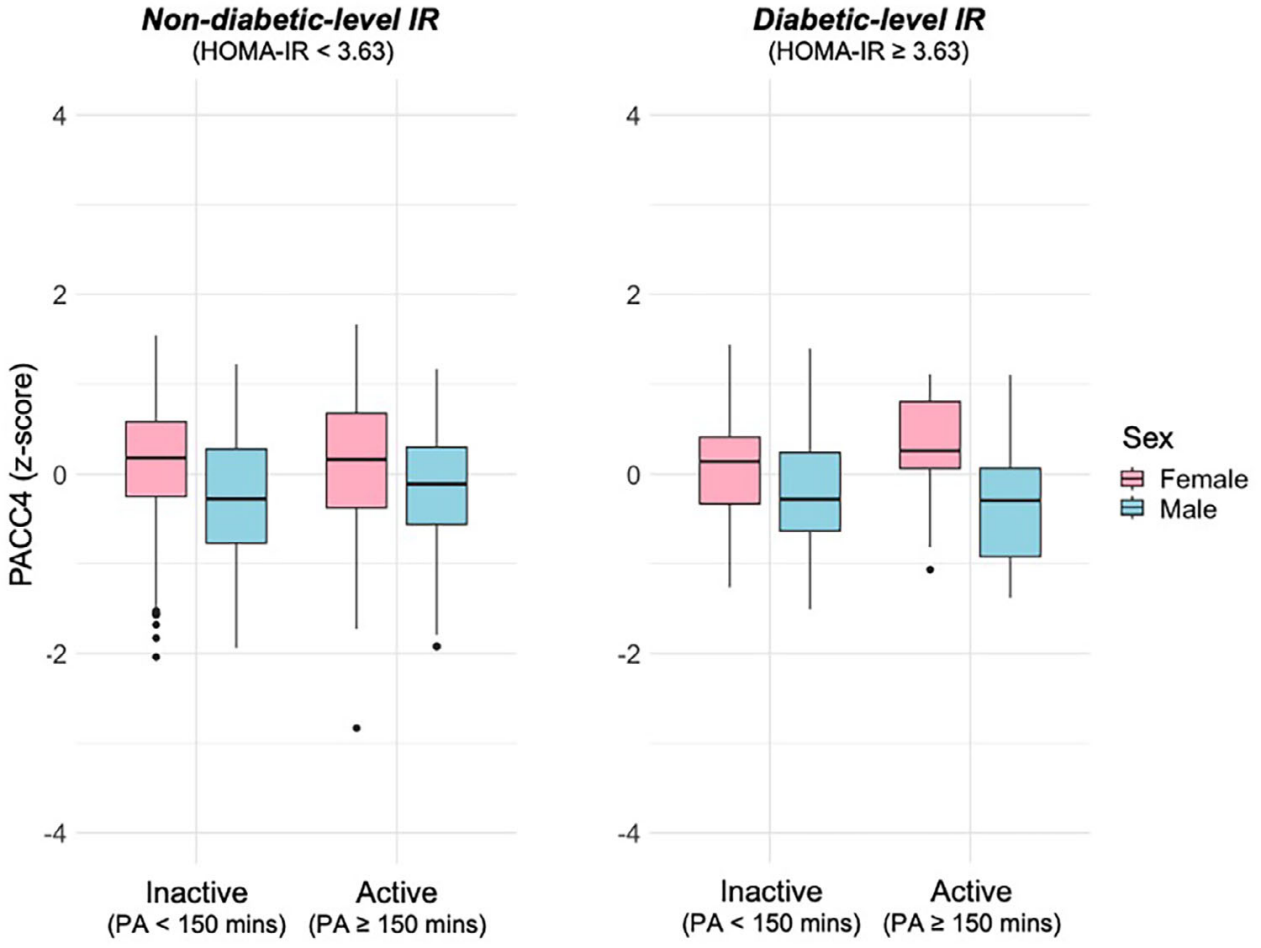# Physical activity and sex modify the influence of diabetic‐level insulin resistance on cognitive function in a cohort enriched with risk for Alzheimer’s disease

**DOI:** 10.1002/alz.089995

**Published:** 2025-01-09

**Authors:** Gabriella M Mamlouk, Kyle J Edmunds, Brianne M. Breidenbach, Clayton Connor McIntyre, Sarah R Lose, Sterling C. Johnson, Ozioma C Okonkwo

**Affiliations:** ^1^ Wisconsin Alzheimer's Disease Research Center, School of Medicine and Public Health, University of Wisconsin‐Madison, Madison, WI USA; ^2^ Wake Forest School of Medicine, Winston‐Salem, NC USA; ^3^ Wisconsin Alzheimer's Disease Research Center, Madison, WI USA

## Abstract

**Background:**

Physical activity (PA) and peripheral insulin resistance are two promising targets for delaying the onset of cognitive impairment in preclinical Alzheimer’s disease (AD). Understanding how these factors interact and whether their influence on cognitive outcomes is sex‐dependent may be crucial for designing effective lifestyle interventions to protect aging brain health. This study examines whether PA and sex modify the relationship between diabetic‐level peripheral insulin resistance and cognitive function in a sample enriched with risk for AD.

**Method:**

Our sample featured n=1,125 cognitively unimpaired participants from the Wisconsin Registry for Alzheimer’s Prevention (WRAP). An index for PA was defined from self‐reported weekly minutes of moderate and vigorous physical activity, where active participants were defined by reporting at least 150 weekly minutes of moderate or vigorous aerobic exercise. Cognitive composite scores were based on neuropsychological examination by trained technicians, and outcomes were standardized to z‐scores across the WRAP database and indexed. The Homeostatic Model Assessment for Insulin Resistance (HOMA‐IR) quantified insulin resistance status, and scores exceeding 3.63 were classified as diabetic‐level. Cross‐sectional linear regression models were assembled in a moderation framework to test the three‐way interaction between PA, sex, and diabetic‐level insulin resistance on cognitive outcomes, and all model covariates included age, years of education, Apolipoprotein E4 (APOE‐ε4) carriage, and familial history of AD.

**Result:**

73.6% of participants had family history of AD and 37.9% were APOE‐ε4 carriers. The sample was 69.9% female, and participants had a mean age of 64.5 ± 7 years and a mean of 16 ± 3 years of education. Models demonstrated significant interactions between PA, sex, and diabetic‐level insulin resistance on three cognitive composite scores: executive function (β=‐0.73, SE=0.30, p=0.017), preclinical Alzheimer's cognitive composite 3 (PACC3) (β=‐0.66, SE=0.27, p=0.014), and PACC4 (β=‐0.50, SE=0.24, p=0.037). In individuals with diabetic‐level HOMA‐IR, the effect of sex on cognitive performance was moderated by PA, with female sex being more strongly linked to higher cognitive performance in physically active subjects.

**Conclusion:**

In older adults at risk for AD, sex differences in cognitive function in individuals with diabetic‐level insulin resistance may depend on physical activity.